# TAAR5 Modulates Sensorimotor Recovery After Spinal Cord Injury

**DOI:** 10.3390/biomedicines14040796

**Published:** 2026-03-31

**Authors:** Anastasiia D. Buglinina, Ekaterina A. Romanyuk, Alexander A. Chesnokov, Sviatoslav I. Milov, Polina Yu. Shkorbatova, Natalia V. Pavlova, Nataliia V. Katolikova, Raul R. Gainetdinov, Daria S. Kalinina, Pavel E. Musienko

**Affiliations:** 1 Neurobiology Department, Sirius University of Science and Technology, Sochi 354340, Russiae.a.r@bk.ru (E.A.R.); svyat.milov@yandex.ru (S.I.M.); 2 Institute of Translational Biomedicine, St. Petersburg State University, St. Petersburg 199034, Russiagainetdinov@spbu.ru (R.R.G.); 3 Pavlov Institute of Physiology, Russian Academy of Sciences, St. Petersburg 199034, Russia; 4 Moscow Center for Advanced Studies, Moscow 127486, Russia

**Keywords:** TAAR5, trace amines, dopamine, spinal cord injury, sensorimotor functions

## Abstract

**Background**: Spinal cord injury (SCI) is a severe pathological condition resulting in persistent motor and sensory impairments. The trace amine-associated receptor 5 (TAAR5) is a potential modulator of central nervous system functions; however, its role in CNS repair remains poorly understood. **Methods**: We comprehensively evaluated the effect of TAAR5 gene knockout on functional recovery following lateral spinal cord hemisection in TAAR5-KO and wild-type (WT) male mice. Sensorimotor recovery after SCI was assessed using the horizontal ladder, grasp, and hindlimb mobility tests. Exploratory and anxiety-like behaviors were evaluated using the open field and elevated plus maze tests before and 5 weeks after SCI. **Results**: TAAR5-KO mice exhibited accelerated recovery of sensorimotor functions, as assessed by joint mobility and grasping tests, compared to WT animals. In contrast, no significant intergroup differences were found in the Horizontal Regular Ladder test, likely due to the task complexity and an insufficient recovery period. Nevertheless, SCI induced elevated anxiety-like behavior regardless of genotype. **Conclusions**: These findings indicate that TAAR5 deficiency exerts a positive modulatory effect on the restoration of specific components of sensorimotor function after SCI. This effect may be mediated through the modulation of dopaminergic neurotransmission and inflammatory processes. The observed beneficial effect of TAAR5 knockout identifies this receptor as a promising target for developing novel therapeutic strategies aimed at improving functional outcomes following spinal cord injury.

## 1. Introduction

Spinal cord injury (SCI) is a severe, life-altering condition characterized by disruption of axonal pathways, leading to temporary or permanent motor, sensory, and autonomic dysfunction below the level of the lesion [1]. SCI can result from traumatic events, such as falls, traffic or sport accidents, as well as from non-traumatic causes like diseases and tumors [2]. The pathophysiological cascade of SCI includes primary and secondary mechanisms. The primary injury refers to the initial mechanical trauma, which disrupts the axonal network and blood vessels. All of it triggers secondary pathological processes, including ischemia, oxidative stress, neuroinflammation, and apoptosis [3].

Currently, spinal cord injury treatment mainly focuses on reducing the mechanical impact on the spinal cord by stabilizing the spine and decompressing it, as well as maintaining blood pressure and respiration [4]. Drug therapy in the acute stage includes the administration of anticoagulants, opiates, nonsteroidal anti-inflammatory agents, short-acting sedatives and various symptomatic treatments for spasticity, neurogenic bowel, etc. [5,6]. All these methods are usually ineffective because they are aimed at supportive therapy, eliminating symptoms or secondary effects, rather than at complete regeneration of nervous tissue or increasing the regenerative capacity of the central nervous system.

Some clinical guidelines [7] include the use of drugs (e.g., methylprednisolone, ganglioside) aimed at secondary damage such as inflammation and oxidative stress, but in recent years there has been increasing evidence of insufficient neurological benefit and severe side effects [5,8]. Among the latest methods, it is worth noting electrical stimulation, especially of the epidural and peripheral nerves. Functional electrical stimulation is increasingly being used to enhance neuroplasticity and improve motor function in some patients [9]. But outcomes of therapy are inconsistent across injury levels and severities, the effects of invasive stimulation such as infections, electrode migrations, and autonomic dysreflexia have also been reported [10]. An additional limitation is that electrical stimulation does not fully reproduce spinal locomotor networks neuromodulation provided by endogenous monoamines [11], whose levels critically decrease after SCI [12]. Descending monoaminergic projections (dopamine, serotonin, epinephrine and norepinephrine) are necessary for the normal functioning of the spinal cord and are important for both sensorimotor and visceral functions, and their damage leads to various disturbances. In clinical guidelines, dopamine is indicated primarily as a chronotropic agent for regulating blood pressure and heart rate in the acute phase of spinal cord injury, while serotonin is used to treat injury-induced depression [5,6]. Several pilot clinical trials have examined the effects of drugs that affect the dopaminergic system. The results were generally inconsistent, although some positive effects were observed with the use of levodopa in combination with Buspirone (a serotonin agonist that also increases the synthesis and availability of dopamine) [13,14]. In another study, a single administration of dopamine caused a sustained increase in triglyceride and ketone body concentrations in individuals with spinal cord injury, which may indicate metabolic stimulation. However, the expected change in cytokine concentrations [15] was not observed, which the authors attribute to the low doses and short duration of the infusion [16]. In animal studies, dopamine has been shown to suppress NLRP3 inflammasome activation and pyroptosis, attenuate neuroinflammation, mitigate secondary damage, and regulate the micturition reflex after spinal cord injury [17,18,19]. Drugs that target the serotonergic system have been shown to be effective not only in animal studies [20,21,22], but there are also positive clinical applications of SSRIs in SCI, such as increasing strength and reducing spasticity and neuropathic pain [23,24].

However, all human clinical trials are conducted cautiously, with small safe dosages and short-term or single-dose administration, as the use of drugs that affect dopamine and serotonin is complicated by numerous side effects. Therefore, the search for targets that gently modulate monoamine neurotransmitters in the CNS and also exhibit regenerative, neuroprotective, and anti-neuroinflammatory properties remains relevant, and trace amines and their receptors may be considered promising candidates. Neuroinflammation plays a pivotal role in both acute and chronic phases of SCI, exerting a dual effect by potentially exacerbating damage while also contributing to enhanced repair [25]. This bidirectional influence has become one of the reasons for the active study of neuroinflammatory processes associated with traumatic injuries of the central nervous system [26].

One element potentially involved in modulating neuroinflammation is the trace amine-associated receptor 5 (TAAR5). Trace amines are a group of endogenous monoamines present at very low (trace) concentrations in the CNS. They modulate the activity of classical monoaminergic systems, including dopaminergic, noradrenergic, and serotonergic pathways. This unique mechanism stems from their structural, metabolic, and physiological similarities to classical monoamine neurotransmitters [27]. It has been demonstrated that certain trace amines can exert a positive effect on inflammation and recovery from traumatic injuries of the CNS [28,29,30]. The primary targets of trace amines are G protein-coupled receptors of the TAAR family [31]. While early research focused on the role of TAARs, particularly TAAR1, in psychiatric contexts, significant progress over the last two decades has demonstrated the broad involvement of this receptor family in various physiological functions. These include modulation of neurotransmitter systems, immune functions, neurogenesis, and regenerative processes [32,33]. Furthermore, emerging evidence suggests that TAARs may indirectly influence sensorimotor functions [34]. Specifically, TAAR5 expressed in the cerebral cortex, limbic system structures, and the spinal cord of neonatal rats, and it is involved in regulating motor coordination, muscle strength, and dynamic balance [35,36,37]. Additionally, the influence of TAAR5 may extend to adult neurogenesis and the regulation of neuronal proliferation: studies in TAAR5-knockout mice have reported a twofold increase in proliferating neurons within the subventricular and subgranular zones—key neurogenic brain regions [32].

Thus, the specific role of TAAR5 in promoting repair and recovery after CNS trauma remains an open question, necessitating additional research. It is unclear how modulating TAAR5 activity affects neural circuit restoration and locomotor function following injury. Investigating these aspects could contribute to the development of novel therapeutic strategies aimed at stimulating neuroplasticity and improving motor outcomes after SCI. This study examines the involvement of TAAR5 in the functional recovery of sensorimotor functions.

## 2. Materials and Methods

### 2.1. Animals

The study utilized adult male mice: wild-type (WT, *n* = 16) and with a knockout of the gene encoding the TAAR5 (TAAR5-KO, *n* = 14). The knockout line was established by crossing heterozygous animals for over ten generations [32]. Prior to and during the experiment, all subjects were housed under standard vivarium conditions with a controlled 12/12 h light/dark cycle, humidity maintained between 45 and 70%, and a temperature of 21 °C. Food and water were provided ad libitum. The experimental procedures were conducted on mice aged 8–10 months with a body weight of 26–30 g. All behavioral testing of mice was performed individually between 12:00 and 19:00 before and after spinal cord injury. To prevent imbalances during the study, mice were randomly assigned to the required groups. The experimental protocols involving animals were designed and performed in compliance with international guidelines for biomedical research using laboratory animals (Directive 2010/63/EU of 22 September 2010) and were approved by the Animal Research Ethics Committee of Saint Petersburg State University (Protocol No. 131-03-14, 23 September 2024). All experimental procedures were performed in accordance with the relevant guidelines and regulations. The present study was conducted in accordance with the ARRIVE guidelines.

### 2.2. Study Design

**Figure 1:**
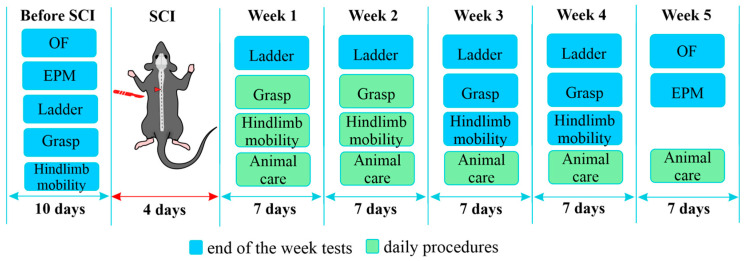
Experimental schedule of surgical procedures and behavioral test timeline. The red triangle marks the area of the spinal cord injury (a lateral hemisection at the T8 vertebral level).

Prior to spinal cord injury (SCI), the open field test and elevated plus maze, also to assess hindlimb motor function the horizontal ladder test, hindlimb mobility test, and grasp test were performed. After SCI, the same tests were conducted for four consecutive weeks at the end of each week, with the exception of the open field and elevated plus maze tests, which were performed at the fifth week (Figure 1). The specific days for conducting the behavioral experiments are described in the relevant section of each test. Hindlimb scoring was performed daily for 10 days and then weekly thereafter. Animal care was provided daily.

### 2.3. Experimental Spinal Cord Injury

A left lateral hemisection spinal cord injury (SCI) model was employed. The lesion was performed at the T8 vertebral level, targeting the T10-T11 spinal cord segments [38]. Lateral hemisection is performed by surgical dissection of one lateral half of the spinal cord, the opposite side remains uncut [39]. With incomplete injury, cardiorespiratory and vegetative stability are maintained and animals do not require intensive care [40,41]. All surgical procedures were conducted under aseptic conditions. Animals were anesthetized using isoflurane (1–2% in oxygen, 0.8 L/min flow rate) and maintained on a heating pad. The depth of anesthesia was routinely assessed by the absence of a tail pinch reflex. The dorsal surgical site was shaved and prepared with 70% ethanol. A midline skin incision was made over the T7–T9 vertebrae, and the underlying paravertebral muscles were dissected bilaterally to expose the spinous processes. The T8 vertebra was identified based on its distinct anatomical landmarks and by systematic counting from the caudal rib attachments. A laminectomy was then performed. A complete left lateral hemisection was subsequently executed using a microscalpel under direct visualization.

Next, muscle and skin layers were sutured using 6-0 Vicryl and 6-0 Ethilon, respectively. Immediately post-surgery, animals received a long-acting antibiotic Bicillin-5 (benzathine benzylpenicillin 24,000 units and benzylpenicillin procaine 6000 units, s/c), 0.5–0.7 mL of 0.9% saline for rehydration, and an analgesic (ketoprofen, 10 mg/kg). To ensure postoperative recovery, mice were administered additional daily subcutaneous injections of saline and ketoprofen for the first 72 h. After the surgery, animals were housed individually. Daily monitoring included assessment of general condition, bladder function and body weight.

### 2.4. Behavioral Assessment

#### 2.4.1. Horizontal Regular Ladder Test

To assess the recovery of sensorimotor function following spinal cord injury (SCI) the horizontal regular ladder test was performed [42]. The apparatus consisted of a horizontal ladder with regular rungs, flanked by transparent sidewalls (50 × 25 cm, elevated above the floor) and a darkened shelter chamber at one end. The distance between rungs was 1 cm. Prior to testing, all animals were acclimated and trained on the apparatus. During testing, mice were placed on the ladder, and the number of errors (slips and misplacements) made by each hindlimb was recorded. Errors occurring immediately after an initial slip or misstep were excluded from the count until the animal fully regained balance and resumed walking. For each trial, a total of 35 steps were evaluated, and the average crossing speed was recorded. Testing was performed pre-SCI and on post-injury days 2, 7, 14, 21 and 28.

#### 2.4.2. Grasping Test

Active flexion of metatarsophalangeal joints of hindlimbs and the ability to lift body weight were assessed using a grasp test [43]. The lower surface of the mouse paws was touched by the rod (7 cm long, 0.2 cm diameter), and movement and sensitivity were scored on a 5-point scale: 4—full weight support and ability to lift the body; 3—ability to support weight but unable to lift the body; 2—ability to grasp the rod and briefly support weight; 1—ability to grasp the rod but unable to support weight; 0—inability to flex digits or grasp the rod. Testing was performed daily for the first 10 days post-SCI and weekly thereafter.

#### 2.4.3. Hindlimb Mobility Test

Mice were placed on a flat horizontal surface, and voluntary joint movements during locomotion were visually assessed [44]. Hindlimb joint function (ankle, knee, and hip) was evaluated using a 4-point scale for each paw separately: 3—no joint dysfunction; 2—near-normal function with minor limitations; 1—markedly impaired function; 0—complete loss of movement. Testing was conducted daily from day 1 to day 10 post-SCI and weekly thereafter until week 4. Scores were summed for analysis.

#### 2.4.4. Open Field

Exploratory activity was assessed using Open field test [45]—an arena (40 × 40 cm) with demarcated central and peripheral zones (OpenScience, Krasnogorsk, Russia). At the beginning of each trial, a mouse was placed in the central zone and allowed to explore freely for 10 min. Sessions were video-recorded and analyzed using EthoVision XT 11.5 software (Noldus, Wageningen, The Netherlands). The resulting video recordings were analyzed for parameters such as distance traveled, average and maximum speed, number of entries, and time spent in the central and peripheral zones. Testing was conducted before and 35–36 days after SCI.

#### 2.4.5. Elevated Plus Maze

Anxiety-like behavior was evaluated in Elevated plus maze [46] using the standard apparatus consisted of two open and two enclosed arms, elevated 30 cm above the floor (OpenScience, Krasnogorsk, Russia). Each mouse was placed in the central zone facing an open arm and allowed to explore for 5 min. All trials were video-recorded and analyzed using EthoVision XT 11.5 (Noldus, Wageningen, The Netherlands). The main parameters assessed were the distance covered, the time spent in open and closed arms, and the number of entries into open and closed arms. Testing was conducted before and 35–36 days after SCI.

### 2.5. SCI Degree Validation

**Figure 2:**
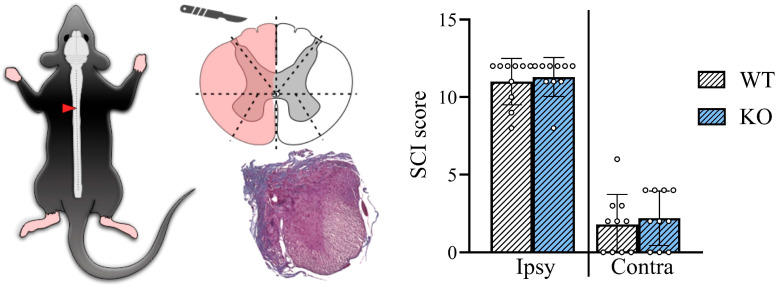
Assessment of the lesion degree after spinal cord injury for the ipsilateral and contralateral sides. The triangle marks the area of the spinal cord injury. Data are presented as mean ± standard deviation, empty circles represent individual data values.

Under deep anesthesia (Zoletil 200 mg/kg + and Xylazine 2 mg/kg, i/p), transcardial perfusion was performed with ice-cold 0.1 M phosphate-buffered saline (PBS, 0.9% NaCl) followed by 10% neutral buffered formalin (BioVitrum, Saint-Petersburg, Russia) for tissue fixation for histological validation of the lesion. The spinal cord segments containing the lesion were dehydrated through a graded series of isopropanol solutions, cleared, and embedded in paraffin blocks. The frontal sections (7 µm) were stained with Mallory’s trichrome to visualize tissue morphology and assess the lesion extent. The validation of the lateral hemisection was performed using a quadrant method. Each frontal section was divided into eight distinct anatomical zones, and the degree of tissue damage within each quadrant was scored on a scale from 0 (intact tissue) to 3 (complete disruption). Scores were summed separately for the ipsilateral (injured) and contralateral sides (Figure 2). Animals with insufficient or excessive hemisection levels and severity, and not comparable to those in the main sample, were excluded from further statistical analysis. Behavioral test results are presented for *n* = 10 WT and *n* = 10 KO mice.

### 2.6. Statistical Analysis

Statistical analysis of the data was performed using GraphPad Prism 9.0 software (GraphPad Software Inc., San Diego, CA, USA). Significant differences were identified using two-way ANOVA (factor 1—time and to compare matched groups) with post hoc Sidak’s multiple comparisons test for Horizontal ladder rung test, Grasp test and Joint mobility assessment, and Kruskal–Wallis test with post hoc Dunn’s multiple comparisons test to compare matched groups for Open field and Elevated plus maze. 6 WT and 4 KO mice were excluded from the final behavioral analysis according to SCI degree validation. Researchers were blinded to the group allocation at all stages of the experiment and data analysis. Sample sizes were determined using G*Power 3.1 (Heinrich Heine University, Düsseldorf, Germany; F tests, ANOVA: repeated measures, within–between interaction; effect size f = 0.40, α = 0.05, power = 0.9, 2 groups, 5 time points, correlation among repeated measures = 0.5, nonsphericity correction ε = 1). An a priori analysis indicated a minimum total sample size of 12 (6 per group). Accordingly, *n* = 10 mice per group was used for all behavioral experiments; post hoc power analysis confirmed an achieved power of 0.997 (total N = 20). Data in charts are presented as Mean ± SD. The significance threshold was set at *p* < 0.05.

## 3. Results

**Figure 3:**
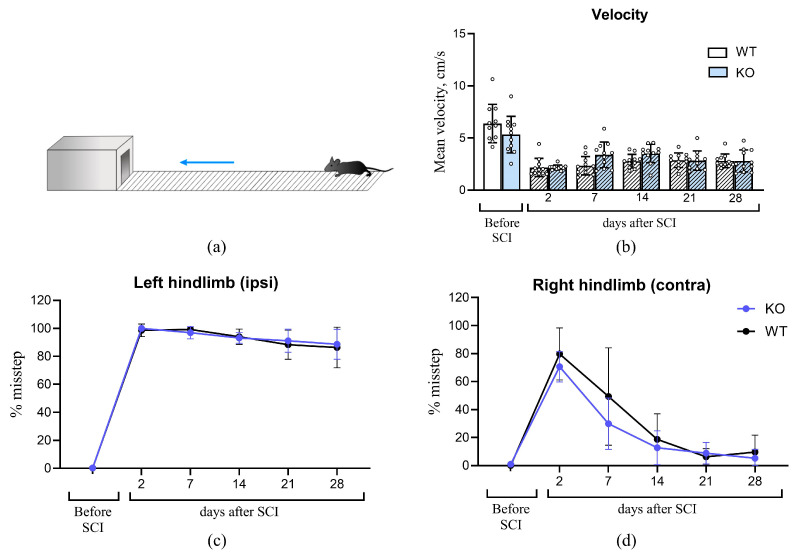
Assessment of sensorimotor function in the horizontal ladder rung test before and after spinal cord injury (SCI). (**a**) Ladder rung test. The blue arrow indicates the direction of mouse movement. (**b**) Mean traversal velocity. (**c**) Percentage of missteps for the left hindlimb. (**d**) Percentage of missteps for the right hindlimb. Data are presented as mean ± standard deviation; empty circles represent individual data values.

The horizontal ladder rung test revealed no differences between the WT and KO groups across various parameters of sensorimotor recovery following SCI (Figure 3). Statistical analysis showed no significant difference in the number of errors made by the left hindlimb (ipsilateral to the lesion) between WT and KO mice (WT: 88.29 ± 10.38%; KO: 91.14 ± 8.24%). Similarly, errors made by the right hindlimb (contralateral to the lesion) were comparable between groups (WT: 6.57 ± 6.03%; KO: 9.71 ± 7.52%). As expected, the mean number of errors for the ipsilateral left hindlimb was higher than for the contralateral right hindlimb. The mean pre-injury traversal speed was 6.52 ± 1.89 cm/s for TAAR5-WT and 5.27 ± 1.84 cm/s for TAAR5-KO mice, with no statistically significant difference between the groups.

**Figure 4:**
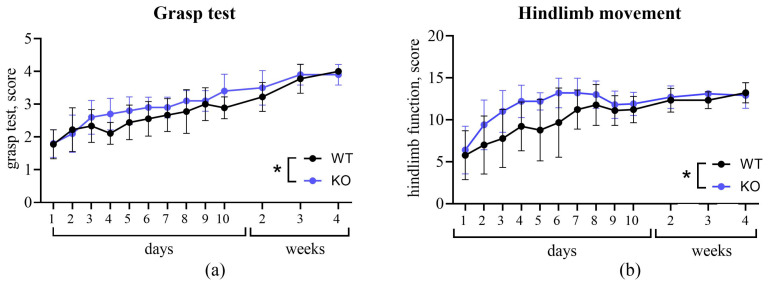
Assessment of sensorimotor recovery dynamics from day 1 to week 4 post-SCI. (**a**) Grasp test. (**b**) Joint mobility assessment. Data are presented as mean ± standard deviation. Two-way ANOVA: * *p* < 0.05 for the main effect of genotype.

In the grasping test (Figure 4a), the KO group demonstrated a higher rate of recovery compared to the WT group (effect of genotype: F(1, 18) = 4.573, *p* = 0.0464). Results from the hindlimb mobility test (Figure 4b) similarly indicated more robust recovery, showing a significant main effect of genotype (F(1, 18) = 4.573, *p* = 0.047).

**Figure 5:**
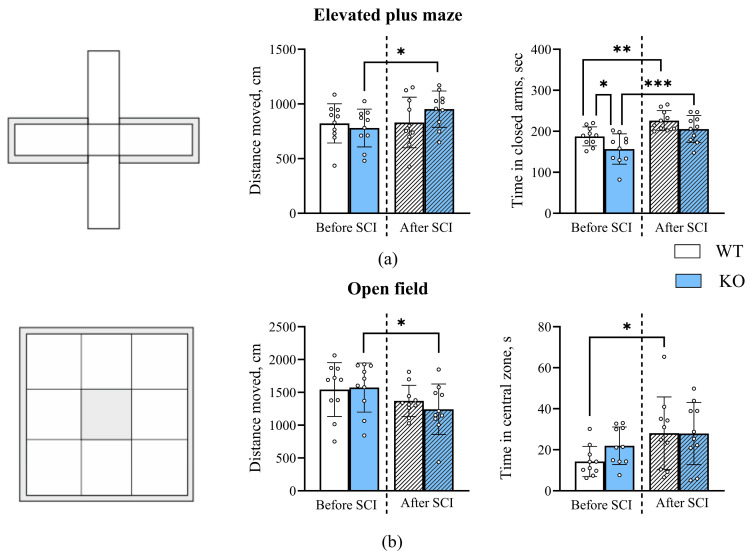
Assessment of anxiety-like behavior and general locomotor activity before and after spinal cord injury. (**a**) Elevated Plus Maze. (**b**) Open Field Test. Data are presented as mean ± standard deviation; empty circles represent individual data values. Statistical analysis was performed using two-way ANOVA with post hoc Fisher’s LSD test: * *p* < 0.05, ** *p* < 0.01, ** *p* < 0.001.

In the Elevated plus maze test (Figure 5a), two-way ANOVA revealed no statistically significant effect of SCI on the total distance traveled. However, post hoc Fisher’s LSD analysis indicated that TAAR5-KO mice traveled a significantly greater distance 5 weeks after SCI compared to their pre-injury baseline (KO before SCI: 780.4 ± 173.2 cm; KO SCI: 952.6 ± 165.3 cm; *p* = 0.05). No significant changes were observed in the wild-type (WT) group. However, SCI had a significant main effect on the total time spent in the closed arms (Two-way ANOVA, F (1, 18) = 33.94; *p* < 0.001), indicating a general increase in time spent in the “safe” zones of the maze post-injury. The main effect of genotype did not reach statistical significance (F (1, 18) = 3.781; *p* = 0.07), though a trend was observed. Post hoc multiple comparisons showed that prior to SCI, WT mice spent significantly more time in the closed arms compared to TAAR5-KO mice (pre-SCI—WT: 183.7 ± 3.551 s; KO: 156.1 ± 3.945 s; *p* = 0.045). After SCI, this difference between genotypes was leveled out (after SCI KO: 215.3 ± 4.191 s; WT: 226.8 ± 5.400 s). Furthermore, a significant post-injury increase in closed-arm time was observed within both the WT (*p* = 0.003) and KO (*p* = 0.001) groups.

In the Open Field Test, two-way ANOVA revealed a statistically significant main effect of SCI on the total distance traveled (F (1, 18) = 7.140; *p* = 0.0155), with a general decrease in locomotor activity following injury (Figure 5b). The main effect of genotype was not significant. Post hoc (Fisher’s LSD) analysis demonstrated that TAAR5-KO mice traveled a significantly shorter distance 5 weeks post-SCI compared to their pre-injury level (KO before SCI: 1608 ± 403.2 cm; KO after SCI: 1244 ± 385.0 cm; *p* = 0.03). In contrast, WT mice showed no significant change in distance traveled after SCI (WT before SCI: 1585 ± 448.6 cm; WT after SCI: 1370 ± 238.8 cm). There were also no statistically significant differences between genotypes either before or after SCI. Analysis of the total time spent in the center of the arena also showed a significant main effect of SCI (F (1, 18) = 6.014; *p* = 0.0246), indicating a general increase in center time post-injury. The main effect of genotype was not significant. Multiple comparisons by post hoc analysis revealed that WT mice spent significantly more time in the center post-SCI compared to their pre-injury baseline (WT pre-SCI: 14.29 ± 7.356 s; WT post-SCI: 28.12 ± 17.68 s; *p* = 0.03). No significant increase was found within the KO group (KO pre-SCI: 22.04 ± 9.275 s; KO post-SCI: 27.95 ± 15.18 s). No significant differences were observed between genotypes before or after SCI.

## 4. Discussion

Spinal cord injury constitutes a serious neurological condition for which current treatment strategies necessitate novel approaches and therapeutic targets. Incomplete spinal cord injury is the most common clinical case [47], and in this study, the lateral hemisection at the thoracic level was used to model incomplete SCI [39]. Such model recapitulates key features of human Brown-Séquard-type spinal cord injury (ipsilateral motor deficit and contralateral sensory changes) [48] and allows comparison of functional recovery between the ipsilateral and contralateral sides, including hind limbs functions [39]. Unlike compression or contusion models, it enables precise disruption of specific spinal cord pathways, thereby improving reproducibility [41].

Concurrent with the rising incidence of spinal cord injury [49], the affected population is aging, reflecting broader demographic trends (Eurostat) [50]. Epidemiological data indicate a steady increase in the average age at injury: for the period 1972–2000, it was about 32 years [49], while for different periods from 2007 to 2025, it was 44 years in the United States [49], 43 years in China [51], and 59 years in Italy [52]. Nevertheless, many preclinical studies continue to employ young animals, thereby overlooking the influence of age on recovery outcomes, despite evidence that older age is associated with diminished regenerative capacity and less favorable prognosis [53,54]. To address this gap, the present study utilized a cohort aged 8–10 months, representative of middle-aged rather than young individuals. This approach is intended to enhance clinical relevance by incorporating age-related biological factors pertinent to the population most susceptible to such injuries. Given the influence of age on recovery outcomes, identifying molecular targets that can effectively promote repair in this demographic is essential. One such target is trace amine-associated receptor 5 (TAAR5).

Trace amines, acting independently or through modulation of the monoaminergic system, can regulate sensorimotor function [35]. Trace amines and their receptors are also involved in immune response regulation [16], which is of significant interest in the context of post-SCI neuroinflammation; however, their potential remains underexplored. TAAR5 expression has been documented in brain regions responsible for motor and vestibular control [36], as well as in some immune cells [55], suggesting its potential role not only in sensorimotor regulation but also in processes of regeneration, repair, and compensation following SCI. This study investigated the influence of TAAR5 on the recovery of sensorimotor functions after spinal cord injury.

According to the obtained data, TAAR5 knockout animals exhibited accelerated recovery of sensorimotor functions. In the voluntary grasp test, TAAR5-KO mice demonstrated more intensive recovery of sensitivity and muscular strength. Similarly, TAAR5-KO mice showed faster restoration of normal hindlimb mobility, indicating a positive effect of TAAR5 knockout on the functional recovery of hindlimbs after SCI.

TAAR5-KO mice have previously been shown to have reduced serotonin levels in certain brain regions [37]. However, rats with a knockout of the gene encoding tryptophan hydroxylase 2 (TPH2, an enzyme that regulates serotonin synthesis in the CNS) exhibit impaired recovery dynamics of muscle tone after SCI [44]. Thus, one might logically expect that reduced serotonin in TAAR5-KO mice would lead to decreased recovery efficacy. However, our study observed the opposite effects. There are several possible reasons for this inconsistency. On one hand, differences in spinal cord serotonin synthesis via mono-enzymatic cells containing AADC (D1-cells) may play a certain role [56]. On the other hand, the primary contribution to improved recovery dynamics in TAAR5-KO mice may stem not from serotonin, but from reduced inflammatory processes and alterations in dopamine. TAAR5-KO mice had higher expression of genes associated with dopaminergic signaling in the striatum and substantia nigra [33], resulting in elevated levels of dopamine and its metabolites in these structures. Regulation of muscle tone is closely linked to D1 and D2 receptors in the striatum and D1 receptors in the substantia nigra. Enhanced dopaminergic neurotransmission has been shown to positively influence muscle tone [57], which could also favorably impact recovery dynamics after SCI. Furthermore, dopamine has been demonstrated to positively affect neuronal proliferation and survival in neurogenic areas of the brain [32], as well as axonal regeneration in spinal cord after injury [19,58,59].

In contrast to other behavioral measures, the results from the Regular Horizontal Ladder test revealed no statistically significant genotype-dependent differences in the number of correct steps or the average traverse velocity, both before and after the induction of SCI. It is important to note that this test is recognized for its sensitivity in detecting subtle sensorimotor integration deficits, surpassing the granularity of voluntary grasping or hindlimb mobility assessments. The evaluation window was limited to 4-week period, which may not have been sufficient to detect significant changes in sensorimotor recovery. Earlier studies have shown that intact knockout animals perform better on sensorimotor coordination tests [36]. However, that study used a significantly more complex, irregular ladder pattern compared to the regular pattern presented here.

SCI consequences are accompanied by a range of affective disorders, including depression and increased anxiety [60,61]. We demonstrated that SCI induces an increase in anxiety-like behavior in the Elevated Plus Maze in both wild-type and TAAR5-KO mice 5 weeks after injury. Notably, TAAR5-KO mice traveled a greater distance in this test, likely associated with some increase in exploratory activity. Pre-SCI results are consistent with previously published data demonstrating a reduced anxiety phenotype in TAAR5-KO mice [37]. The anxiolytic effect of TAAR5 gene knockout is linked to a close interaction between TAAR5 and the serotonergic system [37]. In the Open Field Test, the level of anxiety-like behavior post-SCI in KO mice remained stable and practically unchanged from pre-injury levels. Mice of both genotypes spent more time in the central zone of the arena after SCI, which could suggest reduced anxiety; however, this test primarily reflects general locomotor activity, and a decrease in the total distance traveled was observed. This result differs from the EPM data, likely because in the OFT, mice generally tend to move more and faster, as there are no enclosed “safe” zones to explore [60,62]. Thus, in wild-type mice, TAAR5 may have an inhibitory effect on exploratory activity by influencing emotional behavior via the limbic region of the brain. Under physiological conditions, TAAR5 may exert a tonic inhibitory influence on dopaminergic and serotonergic neurotransmission. Indeed, TAAR5 knockout mice exhibit increased dopamine levels and altered serotonin signaling in the striatum and limbic regions [33,37], suggesting that TAAR5 normally restrains these systems. This inhibitory tone could serve as a homeostatic mechanism to prevent excessive monoaminergic drive, which might otherwise lead to aberrant motor output or emotional dysregulation. However, following spinal cord injury, this same inhibitory influence in wild type mice may become maladaptive by limiting the neuroplastic responses required for functional recovery. In support of this, TAAR5-KO mice exhibit decreased expression of several genes involved in gliogenesis and glial cell differentiation, accompanied by increased expression of genes associated with neuronal differentiation, axonogenesis, and synaptogenesis in striatum [33], which is considered as another adult neurogenesis zone [63].

### Limitations of the Study

Although TAAR5 has been identified as the most evolutionarily conserved subtype within the TAAR family across all mammalian species studied to date, direct translation may be difficult [64]. TAAR5 has been identified as a pseudogene in some primate species [65], and its ligand-binding affinities are variable. For instance, trimethylamine and dimethylamine display no agonistic activity at TAAR5 in most primates, while they activate TAAR5 in mice, rats, dogs, and humans [66,67]. Moreover, dimethylethylamine stimulates Gs signaling while downregulating the Gq/11 pathway for mTAAR5, an effect not observed for the hTAAR5. In contrast to m TAAR5, application of 3-iodothyronamine (3-T_1_AM) to hTAAR5 resulted in a significant reduction in basal inositol phosphate (IP_3_) production and MAP kinase signaling [68]. The mouse hemisection model allows us to study fundamental mechanisms of neuroplasticity and regeneration that may be common across mammals, but anatomical and immunological differences may also contribute to recovery processes [69]. In addition, constitutive knockout, in particular the TAAR5 gene, may modify the animal physiology, adaptation and compensation mechanisms, resulting in CNS rearrangements during development [70], that also may have influence on the recovery and regeneration processes after injury. Our findings open the door to pharmacological modulation of TAAR5 (e.g., using antagonists) as a strategy for enhancing post-injury recovery. However, caution is needed when extrapolating, given the differences in spinal cord anatomy and physiology between species.

## 5. Conclusions

This study demonstrates that TAAR5 gene knockout exerts a modulating influence on the recovery process following spinal cord injury. Despite the absence of pronounced differences in complex coordinated motor acts (the Regular Horizontal Ladder test), mice deficient in TAAR5 showed significantly enhanced recovery in parameters such as grasp assessment and the range of active joint movements. This suggests that TAAR5 is involved in the pathologic mechanisms that limit neuroplasticity and regeneration in the injured spinal cord. Potential mechanisms underlying the observed positive effect may include TAAR5-mediated modulation of dopaminergic and serotonergic systems, as well as its potential influence on neuroinflammatory processes. Thus, TAAR5 inhibition could be considered a novel and promising direction for developing therapies aimed at improving functional outcomes after spinal cord injury. Further research is required to fully elucidate the cellular and molecular mechanisms underlying the observed phenomenon.

## Data Availability

The data presented in this study are available on request from the corresponding author due to a continuation of analysis of the ongoing research project.
